# Host plant genetic control of associated fungal and insect species in a *Populus* hybrid cross

**DOI:** 10.1002/ece3.6266

**Published:** 2020-04-27

**Authors:** Sandra J. Simon, Timothy J. Tschaplinski, Jared M. LeBoldus, Ken Keefover‐Ring, Muhammad Azeem, Jin‐Gui Chen, David Macaya‐Sanz, William L. MacDonald, Wellington Muchero, Stephen P. DiFazio

**Affiliations:** ^1^ Department of Biology West Virginia University Morgantown West Virginia; ^2^ Biosciences Division and Center for Bioenergy Innovation Oak Ridge National Laboratory Oak Ridge Tennessee; ^3^ Forest Engineering, Resources & Management Oregon State University Corvallis Oregon; ^4^ Botany and Plant Pathology Oregon State University Corvallis Oregon; ^5^ Department of Botany University of Wisconsin-Madison Madison Wisconsin; ^6^ Department of Geography University of Wisconsin Madison Wisconsin; ^7^ Department of Chemistry COMSATS University Islamabad Abbottabad Pakistan; ^8^ Division of Plant and Soil Sciences West Virginia University Morgantown West Virginia

**Keywords:** community genetics, comparative genomics, plant–biotic interactions, *Populus*, tandem duplication

## Abstract

Plants employ a diverse set of defense mechanisms to mediate interactions with insects and fungi. These relationships can leave lasting impacts on host plant genome structure such as rapid expansion of gene families through tandem duplication. These genomic signatures provide important clues about the complexities of plant/biotic stress interactions and evolution. We used a pseudo‐backcross hybrid family to identify quantitative trait loci (QTL) controlling associations between *Populus* trees and several common *Populus* diseases and insects. Using whole‐genome sequences from each parent, we identified candidate genes that may mediate these interactions. Candidates were partially validated using mass spectrometry to identify corresponding QTL for defensive compounds. We detected significant QTL for two interacting fungal pathogens and three insects. The QTL intervals contained candidate genes potentially involved in physical and chemical mechanisms of host–plant resistance and susceptibility. In particular, we identified adjoining QTLs for a phenolic glycoside and *Phyllocolpa* sawfly abundance. There was also significant enrichment of recent tandem duplications in the genomic intervals of the native parent, but not the exotic parent. Tandem gene duplication may be an important mechanism for rapid response to biotic stressors, enabling trees with long juvenile periods to reach maturity despite many coevolving biotic stressors.

## Graphical abstract

This work focuses on elucidating the host genetic drivers of community composition in a hybrid *Populus* family. We mapped quantitative trait loci for pathogenic fungi and leaf modifying insects in plantations and connected these to genomic intervals of the two host species. We found that tandemly duplicated genes were significantly enriched in the intervals and that these genes have functions that are consistent with rapid host co‐evolution with the interacting organisms.

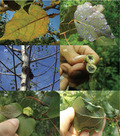

## 1 INTRODUCTION

In natural ecosystems, the dynamics of plant interactions with other living organisms are complex. This is especially true for organisms that rely on plants for shelter, nutrition, and reproduction, such as fungi and insects (Chisholm, Coaker, Day, & Staskawicz, 2006; Panda & Khush, 1995). Although fungi and insects can provide some of the same useful services in return, those that do not can be extremely harmful to plant productivity. To mitigate the effects of biotic stress, plants employ a diverse set of defense mechanisms including chemical, protein‐derived molecules, and physical barriers (Panda & Khush, 1995). Insects and fungi must develop strategies in parallel to overcome these obstacles to survive (Chisholm et al., 2006; Mello & Silva‐Filho, 2002). The theory of gene‐for‐gene coevolution has frequently been used to describe this host plant genetic relationship to its arthropod and fungal communities (Chisholm et al., 2006; Ehrlich & Raven, 1964; Mello & Silva‐Filho, 2002; Thompson, 1988).

The gene‐for‐gene theory suggests a very simple dynamic for the genetic interactions that occur between two species. A gene in the host plant that is important in biotic relationships has a corresponding, coevolving gene from a pathogen/insect which can lead to resistance or susceptibility depending on the life history of the pathogen/insect (Flor, 1971; Friesen, Meinhardt, & Faris, 2007). Much of the evidence for these interactions has been found in crop systems where plant species often have dominant, single‐gene resistance to feeding (Thompson, 1988). For example, there are over twenty different genes in wheat (*Triticum aestivum* L.) that each confers resistance to the Hessian fly, *Mayetiola destructor* (Thompson & Burdon, 1992). Exposure of Hessian fly populations to these resistant varieties of wheat created selection pressure that led to increased virulent gene combinations in the pest (Gallun, 1977; Panda & Khush, 1995). Similarly, in plant–fungal systems breeding for dominant resistance in cereal crops resulted in new selective forces that increased virulent gene frequencies in *Puccinia* spp. cereal rusts (Chen, 2005; Pretorius, Singh, Wagoire, & Payne, 2000). This in turn can lead to an evolutionary arms race between plants, insects, and fungi with the continual development of mechanisms to overcome both genetic defenses and virulent attacks (Bergelson, Kreitman, Stahl, & Tian, 2001; Thompson & Burdon, 1992).

The relationships of host plant genetics and biotic association can also be more complex than these crop breeding systems suggest, and they can leave a lasting impact on genome structure (Lefebvre & Chèvre, 1995). Host plant and biotic associations can lead to the expansion of gene families responsible for the host plant response to biotic stress. For example, the Kunitz protease inhibitors (KPIs) in *Populus* are important in defense responses against insects by inhibition of herbivore digestion (Haruta, Major, Christopher, Patton, & Constabel, 2001; Major & Constabel, 2008). The KPI gene family has greatly expanded in response to insect attack through tandem duplication events (Philippe, Ralph, Külheim, Jancsik, & Bohlmann, 2009). Similarly, plant resistance (*R*) genes, which are important in the defense response of plants to fungal pathogen attack, have also expanded through tandem and segmental duplication events due to biotic pressures (Hulbert, Webb, Smith, & Sun, 2001; Leister, 2004). Analyzing how the genome is structured in the host plant when it associates with fungi and insects is important for studying these relationships and understanding the complexities of their genetic interactions.

Given their rapid growth and vegetative reproduction, *Populus* species have become a focus for research into biofuel production making them a valuable commercial crop (Meilan et al., 2002; Stanton, Neale, & Li, 2010; Taylor, 2002). *Populus* has also become an important genetic model for research into a wide variety of ecological and adaptive traits (McKown et al., 2014), including interactions with the biotic community (Crutsinger et al., 2014; Whitham et al., 2006). In particular, interspecific hybrids of *P. trichocarpa* × *P. deltoides* segregate for a wide variety of traits including resistance to insect and fungal attack (Newcombe, 1998; Newcombe & Ostry, 2001). Such hybrid family crosses can be used to identify regions of the genome that are important in mediating biotic stress.

In this study, we investigated the genome composition of loci associated with insect and fungal species in an interspecific *Populus* family. We surveyed insects and fungal pathogens in a *P. deltoides* × *P. trichocarpa* pseudo‐backcross family and used quantitative trait locus (QTL) analysis and comparative genomics to address three main questions: (a) Is there heritable, host genetic control of fungal and insect species? (b) What protein domains and gene ontology terms are enriched in the QTL intervals in the genomes of each *Populus* species? (c) What candidate genes in the intervals are unique to each species when comparing the *P. trichocarpa* and *P. deltoides* genomes?

## 2 METHODS

### 2.1 QTL mapping pedigree

The 52–124 family was developed by crossing a male *Populus deltoides*, ILL‐101 from southern Illinois, with a female *Populus trichocarpa* clone, 93–968 from western Washington State. The F_1_ clone, 52–225, was then crossed with a male *Populus deltoides* clone, D124 from Minnesota, to generate the final 749 progeny in the pseudo‐backcross population.

### 2.2 Field site descriptions

Clonal cuttings of the 52–124 progeny were obtained from the University of Minnesota and the University of Florida. The individuals were propagated in the West Virginia University (WVU) greenhouses in March 2006. A hay production field was tilled and disked at the WVU Agronomy farm (39°39′32″N 79°54′19″W) in Morgantown, West Virginia for planting. In July 2007, the rooted cuttings were planted with two clonal replicates for each of 749 genotypes at 2 m × 2 m spacing in an interlocking block design. The plantation was thinned in December of 2008 by removing 50% of the trees in a diamond fashion resulting in 2.83 m × 4 m spacing of the remaining trees. A plantation of the same family cross was established at the Westport Research Station in the Columbia River floodplain (46°07ʹ58.8ʺN 123°22ʹ05.0ʺW) in April 2016. The trial was planted with a total of 339 progeny replicated in a randomized complete block design with three blocks.

### 2.3 SNP genotyping and genetic map construction

SNP loci were selected from whole‐genome resequencing data generated for the parent trees of the pedigree, focusing on loci that were fixed for different alleles in *P. deltoides* and *P. trichocarpa*. Sequencing was performed on the Illumina GAII system with single‐end read lengths of 75 bp and a total depth of ~35× on average. Reads were aligned using MAQ, and SNPs were called using Samtools mpileup with a minimum quality of 30 and a minimum depth of 5 reads per allele, and a subset of loci was confirmed by Sanger sequencing (Slavov et al., 2012). Polymorphic loci were selected that were maternally informative for *P. trichocarpa*. These were incorporated into an Illumina Infinium Bead Array which was used to genotype 3,568 SNP loci in 692 of the progeny. These data were then used to create the genetic map which was composed of 19 linkage groups corresponding to 19 *Populus* chromosomes. Genotyping and map construction are described in more detail elsewhere (Muchero et al., 2015).

### 2.4 Family 52–124 parent and progeny phenotyping

In order to identify regions of the *Populus* genome associated with biotic stress, a variety of fungal pathogens and insect herbivore species was surveyed in the WVU Morgantown and Westport Oregon plantation sites. *Melampsora* sp. rust was identified visually by local pathologist Dr. William MacDonald. *Sphaerulina musiva* was identified by sequencing of the ITS region (Verkley, Quaedvlieg, Shin, & Crous, 2013). Insect identification was completed using insect morphological features, feeding symptoms, and known hosts/species distributions for *Phyllocolpa* sp. (Kopelke, 2007; Smith & Fritz, 1996), *Mordwilkoja vagabunda* (Ignoffo & Granovsky, 1961a, 1961b), and *Pemphigus populitransversus* (Bird, Faith, Rhomberg, Riska, & Sokal, 1979; Faith, 1979).

**FIGURE 1:**
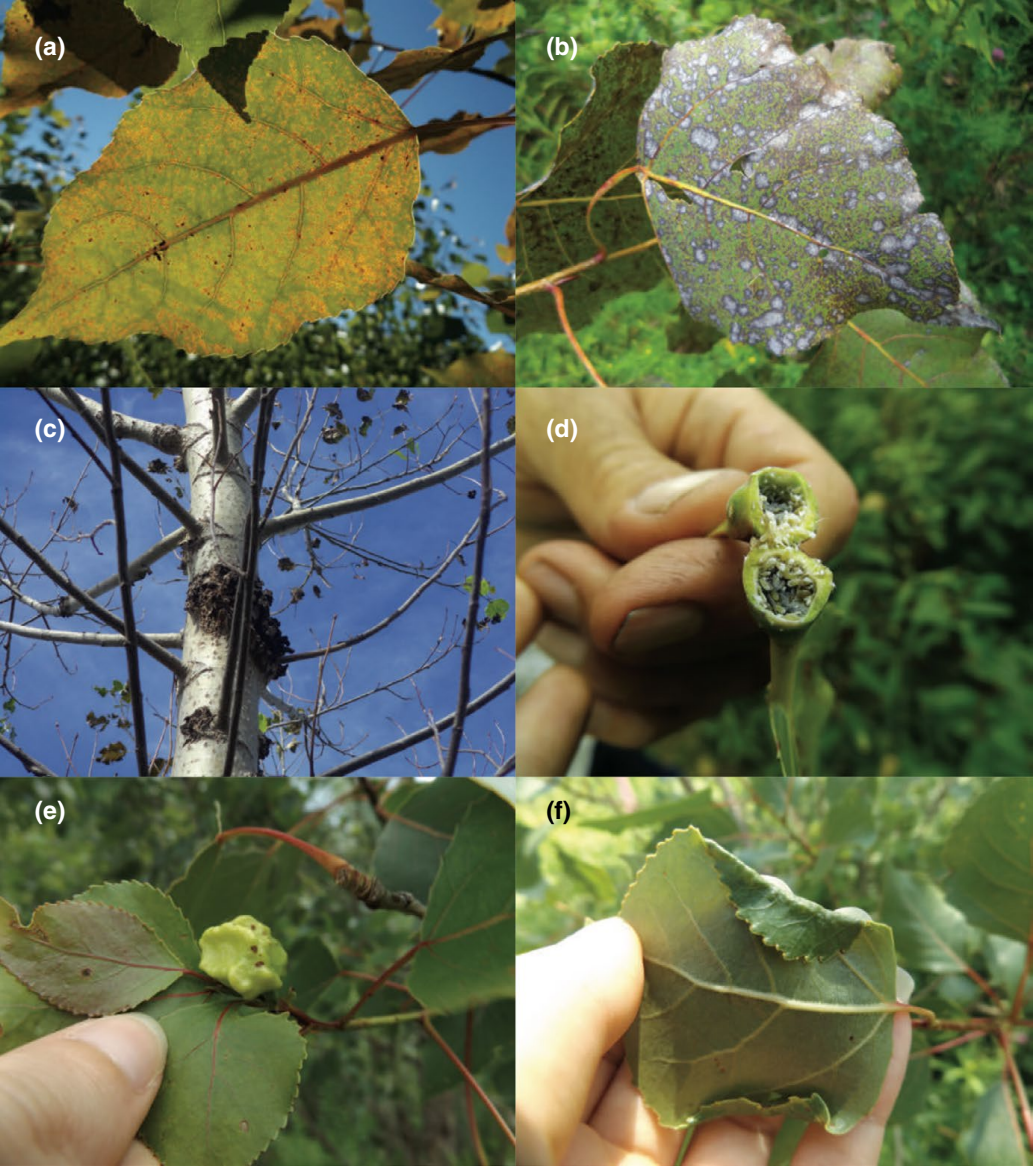
Biotic phenotype symptoms observed on trees including (a) leaf symptoms of *Melampsora* sp. fungal leaf rust, (b) leaf spot symptoms of the *S. musiva* fungus, (c) *S. musiva* canker symptoms, (d) branch gall created by the *M. vagabunda* aphid (e) petiole gall created by the *P. populitransversus* aphid, and (f) *Phyllocolpa* sp. leaf folding gall

*Melampsora* sp. leaf rust (Figure 1a) and *S. musiva* fungal leaf spot symptoms (Figure 1b) were scored on a 0–3 scale of disease severity, with 0 the indicating absence of symptoms and 3 indicating high degree of pathogen leaf damage, in the 2008 growing season for all 1,353 tree canopies. In the fall of 2014, stem canker symptoms caused by the same fungus *S. musiva* (Figure 1c) were scored on a 0–5 disease severity scale for a subset 498 unique genotypes and a total of 580 trees. Upon further examination of field conditions for *S. musiva* disease severity, it was determined that none of the progeny displayed complete resistance. The original 0–5 scale was binarized with scores from 2.5–5 scaled to 1 and 0–2 scaled to 0. The new scale indicated the progression of infection with 1 specifying severe canker development and 0 indicating less aggressive canker symptoms.

Two abundant species of galling aphid were also surveyed during the 2016 growing season. To equalize the biomass surveyed for the aphid insect observations on each tree, branches of equal diameter were selected for insect counts and the remaining branches in the canopy were not surveyed. Tree canopy presence or absence for the petiole galling aphid *P. populitransversus* (Figure 1d) and the branch galling aphid *M. vagabunda* (Figure 1e) was recorded for 201 unique genotypes and a total of 218 trees. For all of the damage scores in every survey year, 0 indicated a complete absence of fungal or insect presence in tree canopy.

In early August 2017, at the Westport site full canopies were scored by counting galls of the leaf‐folding sawfly *Phyllocolpa* sp. (Figure 1f), on 534 unique genotypes and a total of 1,020 trees. To estimate productivity of individuals and confirm that availability of resources did not drive insect attraction or feeding, main stem diameter in millimeters was recorded for all trees to be used as a covariate in the analysis.

### 2.5 Leaf metabolites

The metabolomic profiles for 211 genotypes of family 52–124 were determined for leaves (leaf plastochron index 7 ± 2) sampled on 14 September 2006 at a USDA‐FS field site near Grand Rapids, MI from plants in their first growing season in the field. Leaves were quickly collected and flash‐frozen on dry ice, prior to shipping back to Oak Ridge National Laboratory, where ~200 mg fresh weight per sample was extracted in 80% ethanol (aqueous), as described in Zhao et al. (2010), and analyzed for the major aromatic metabolites following trimethylsilylation and analysis by gas chromatography–mass spectrometry (GC‐MS) using electron impact ionization (70 eV). The analytical and data extraction protocols were similar to that reported previously in Weston et al. (2015). The major phenolic metabolites quantified included salicortin, α‐salicyloylsalicin, salicyl‐salicylic acid‐2‐O‐glucoside, salirepin (gentisyl alcohol 2‐O‐glucoside), gentisic acid 5‐O‐glucoside, 1,2,4‐benzenetriol, and gentisyl alcohol 5‐O‐glucoside.

### 2.6 Statistical analysis

#### 2.6.1 Within‐family broad‐sense heritability (*H* ^2^) calculation

To prevent arbitrary score bias, *Melampsora* sp. and *S. musiva* values were normalized to have a mean of 0 and standard deviation of 1 (*score.transform* function of the *CTT* R package) by applying the inverse of the cumulative distribution function of the normal distribution to the sample percentile score (Gianola & Norton, 1981). Surveys of the *Melampsora* sp. leaf rust, *S. musiva* leaf spot, and counts of the *Phyllocolpa* sp. galls had within‐garden microsite variation removed using thin‐plate spline regression (*fields* R package). Residuals of the spatial correction were added to the mean of each survey dataset for each tree observation to spatially correct and rescale the values. These corrected values were then used to calculate the proportion of variance in the fungal and insect distributions that were due to genotype using a linear‐mixed model (*lmer* function of the *lme4* R package) with insect counts and fungal scores as the response, tree genotype as the predictor, and either competing fungus score or stem diameter biomass estimates as a covariate where applicable. Broad‐sense heritability was calculated as $H^{2}=\delta_{g}^{2}/\left(\delta_{g}^{2}+\delta_{e}^{2}\right)$ , where $\delta_{g}^{2}$ is the genetic variance due to genotype, and $\delta_{e}^{2}$ is the residual variance. Rapid, simulation‐based exact likelihood ratio tests were used to evaluate the significance of variation due to genotype for each linear model (*exactRLRT* function of the *RLRsim* R package). SAS software version 9.4 (2013) was used to test for the normality of all datasets, and for all subsequent statistical analyses, transformations were conducted when necessary. Finally, best linear unbiased predictors (BLUPs) were extracted from these models to use in the QTL analysis.

#### 2.6.2 Quantitative Trait Loci (QTL) analysis

The R software package *R/qtl* (Broman, Wu, Sen, & Churchill, 2003) was used for all QTL analyses described below. Composite interval mapping (CIM) was used to associate *Melampsora* sp. leaf rust, *S. musiva* leaf spot, and *Phyllocolpa* sp. to QTL positions (*cim* function). An additional CIM QTL analysis was conducted for fungi surveyed in 2008 to further evaluate potential competitive bias of co‐occurring fungal pathogens. This was done by subsetting individuals out of the full dataset to exclude trees with symptoms of both pathogens, leaving us with 90 individuals that only showed *Melampsora* sp. symptoms and 434 individuals only infected with *S. musiva* leaf spot. Single QTL mapping was used to associate the binary scores for the *S. musiva* canker, *M. vagabunda,* and *P. populitransversus* to QTL positions (*scanone* function). The method used for both mapping approaches was the expectation–maximization (EM) algorithm. Estimation of QTL interval significance was completed by performing 1,000 permutations. Intervals with logarithm of odds (LOD) scores that were above the *p*‐value threshold (alpha = 0.05), as determined from the permutation tests, were selected for further analysis. The percent variance explained by significant markers for fungal and insect surveys, that were mapped using CIM, were calculated by extracting significant marker positions and creating a fit QTL model (*fitQTL* function). The positive allele contributing to an increase in susceptibility to fungi and insects was found by generating effect plots for each phenotype and its significant marker position (*effectplot* function).

### 2.7 Physical genome intervals

Physical genome intervals in the *P. trichocarpa* genome (v3.0) were examined for each significant QTL for biotic associations. The intervals were defined as 1 Mb regions centered on the marker with the highest LOD score. Fixed physical genome sizes were used rather than intervals defined based on LOD scores due to the large variation in magnitude of LOD observed for the significant QTL. For example, intervals of 1 LOD centered on the QTL ranged in size from 169 to 4,620 kb. Much of this variation was likely due to variation in marker density and local recombination rates, in addition to phenotyping and genotyping error. We believe that a fixed 1 Mb interval is a more consistent and conservative approach given the size of the family and the variation in strength of the QTL (Yin, DiFazio, Gunter, Riemenschneider, & Tuskan, 2004). On average, this represents approximately 6.34 cM, based on a total map size of 2,617 cM and a total assembled genome length of 420 Mb.

**FIGURE 2:**
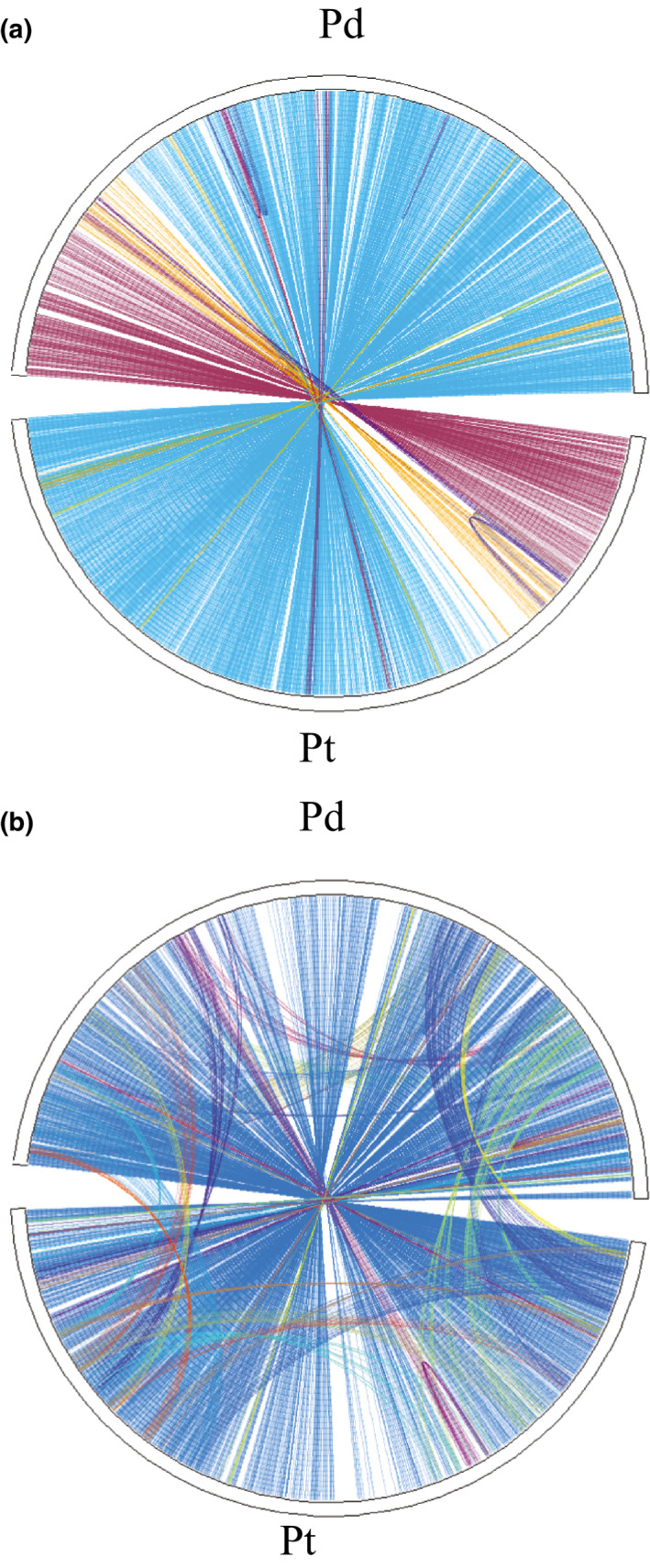
Collinear genes in *P. deltodies* (Pd) and *P. trichocarpa* (Pt) chromosomes, based on MCScanX alignments. (a) Chr02; (b) Chr05

Orthologous intervals were identified in the *P. deltoides* clone WV94 reference genome (v2.1) obtained from Phytozome (Goodstein et al., 2012). Orthology was determined using a combination of protein sequence conservation and synteny using MCScanX (Wang et al., 2012). Briefly, all proteins were compared in all‐vs‐all searches using blastp both within genomes and between genomes. These were then chained into collinear segments using the MCScanX algorithm. Orthologous segments were identified based on the presence of large numbers of gene pairs in collinear order with high sequence identity (median blastp *E* score < 1*e*−180; Figure 2). Synonymous (Ks) and nonsynonymous (Ka) nucleotide substitution rates were calculated using the Bioperl DNAstatistics module (Stajich, 2002; Table S1), domain composition (Table S2), and Gene Ontology (GO) terms (Table S3) were obtained for each genome from Phytozome (v12.1). Intervals were customized for the grandparents of the pseudo‐backcross progeny (clones 93–968 for *P. trichocarpa* and D124 for *P. deltoides*) by converting the respective reference genome based on alignment of short‐read sequences derived from each species. Specifically, we generated 243 and 248 million 250 bp paired‐end Illumina HiSeq sequences for 93–968 and D124, respectively. This yielded an average coverage of ~150× per genome. These were aligned to the respective reference genome for each species (Nisqually v3.0 for *P. trichocarpa* and WV94 v2.0 for *P. deltoides*) using bwa mem with default parameters. SNPs and small indels were identified using samtools *mpileup* and bcftools *call* with default multiallelic variant settings (Li, 2011; Li et al., 2009), and sequence depth was extracted using vcftools (Danecek et al., 2011). Sequences were converted using the vcftools utility vcf‐consensus. Genes with no coverage in the alignments were excluded from the intervals for each species.

### 2.8 Tandem duplications

**FIGURE 3:**
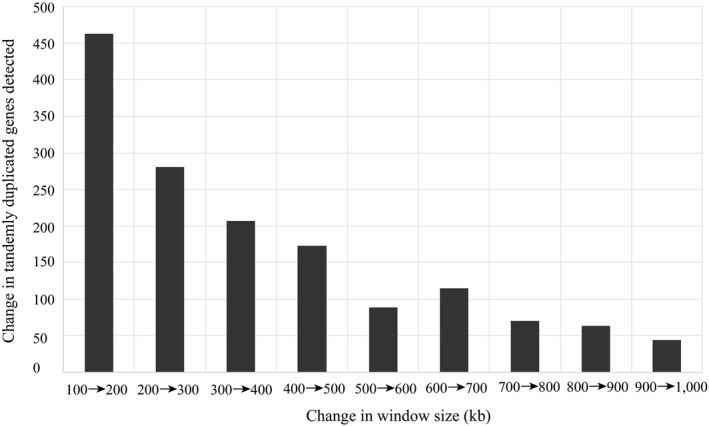
Change in number of tandemly duplicated genes discovered with increasing window size

Tandemly duplicated genes were identified using all‐vs‐all blastp searches within each genome for biotic stress‐associated intervals (Table S4). Genes with blastp *E* scores < 1e−180 that were located within 500 kb of one another were considered to be recent tandem duplications. The window size was determined by testing a range of values and choosing a window size at which the number of newly discovered tandem duplicates began to decline (Figure 3). The *E* score cutoff was chosen because *P. trichocarpa* and *P. deltoides* orthologs also have a median blastp E score in this range, suggesting that these tandem duplications mostly occurred after these species diverged from a common ancestor. This should focus the analysis primarily on genes that are recently duplicated and therefore potentially differentially duplicated between the species. The QTL intervals were tested for significant enrichment of tandem duplicates by using a Monte Carlo simulation. Sets of contiguous genes equal in number to those contained in each QTL interval were randomly selected from the whole genome, and the number of sampled tandem duplications was counted for each iteration. This was repeated 10,000 times, and the observed number of tandem duplicates was compared to the simulated distribution to derive an empirical *p*‐value.

## 3 RESULTS

### 3.1 Heritability of fungal and insect associations

Clonal repeatability (or within‐family broad‐sense heritability) was estimated for each categorical survey trait (Table 1). There was a significant host plant genetic contribution of moderate effect controlling the association of *S. musiva* leaf spot disease severity (*H* ^2^ = 0.250, *p*‐value < .0001). There was a strong effect of host plant genetics on associations of *Melampsora* sp. disease severity (*H* ^2^ = 0.609, *p*‐value < .0001) and the continuous count of leaf‐folding galls of *Phyllocolpa* sp. (*H* ^2^ = 0.391, *p*‐value < .0001).

**TABLE 1:** Linear‐mixed model output for biotic surveys

Fungus/insect	*H* ^2^	Genetic variance	Error variance	RL ratio	*p*‐Value
*Melampsora* sp. leaf rust	0.609	0.271	0.174	294	<.0001
*S. musiva* leaf spot	0.250	0.153	0.459	32.0	<.0001
*Phyllocolpa* sp.	0.391	11.9	18.5	129	<.0001

Note

Broad‐sense heritability (*H* ^2^) denotes the contribution of all host plant genetic factors to total variance in the biotic phenotype. R package RLRsim exactRLRT function was used to test significance of effects in mixed model.

### 3.2 QTL mapping of fungal and insect surveys

**FIGURE 4:**
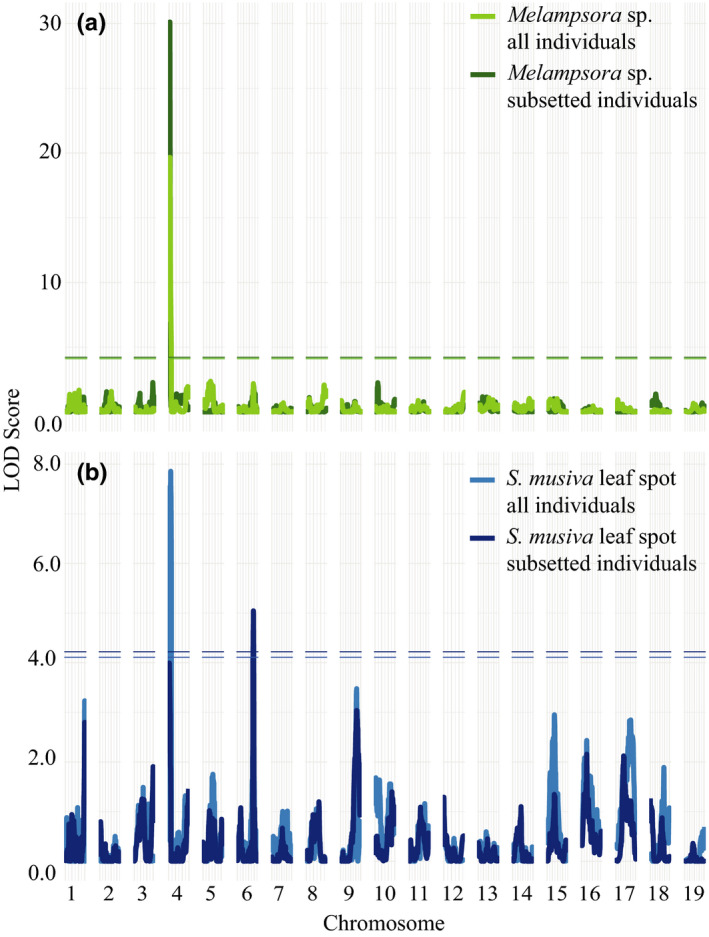
QTL interval plots showing peaks across the genome that associate with 2008 biotic surveys. Lines on the plots indicate *p*‐value thresholds as determined by running 1,000 permutations of mapping model for (a) *Melampsora* sp. model with all individuals and clones subsetted to exclude individuals with *S. musiva* infection and (b) *S. musiva* leaf spot model with all individuals and clones subsetted to exclude individuals with *Melampsora* sp. infection

Four QTL intervals containing a total of 38 markers were significantly associated with the abundance of fungal pathogens (Table 2). An overlapping QTL on Chr04 (Figure 4a,b) was associated with both *Melampsora* sp. (marker position = 3.50687, *p*‐value = .001) and *S. musiva* leaf spot disease severity (marker position = 15.4912824, *p*‐value = .026) for analysis that included all clones. The marker with the highest LOD score explained 54.1% of the variance in the disease severity of *Melampsora* sp. within the Chr04 QTL interval, whereas the top marker within the overlapping interval only explained 3.31% of the variance in the disease severity of *S. musiva* in the analysis with all individuals.

**TABLE 2:** Summary of QTL permutation test output

Model	Chrom.	LOD score	*p*‐Value	% Variance	+ Allele	Garden
*Melampsora* sp. leaf rust all individuals	Chr04	19.7	.001	52.5	D	Morgantown
*Melampsora* sp. leaf rust subsetted individuals	Chr04	30.1	<.0001	57.9	D	Morgantown
*S. musiva* leaf spot all individuals	Chr04	7.80	.001	5.29	D	Morgantown
*S. musiva* leaf spot subsetted individuals	Chr06	4.60	<.0001	4.77	T	Morgantown
*S. musiva* canker	Chr16	4.34	.022	NA	T	Morgantown
*M. vagabunda*	Chr05	5.39	.001	NA	D	Morgantown
*P. populitransversus*	Chr03	2.70	>.05	NA	NA	Morgantown
*Phyllocolpa* sp.	Chr10	6.60	.015	9.65	T	Westport
*Phyllocolpa* sp.	Chr13	4.99	.045	8.82	T	Westport
Gentisyl alcohol 5‐O‐glucoside	Chr17	12.2	<.0001	25.760	T	Grand Rapids
Gentisyl alcohol 5‐O‐glucoside	Chr10	10.3	<.0001	23.104	T	Grand Rapids
Gentisyl alcohol 5‐O‐glucoside	Chr14	5.96	.0100	7.296	T	Grand Rapids

Note

Percent variance in surveys for insects and fungi explained by significant marker indicated for composite interval mapping models. Positive (+) allele specifies genotype at significant interval that results in an increase in susceptibility. D indicates progeny are homozygous for *P. deltoides* alleles, and T indicates progeny are heterozygous for *P. deltoides* and *P. trichocarpa* alleles.

**FIGURE 5:**
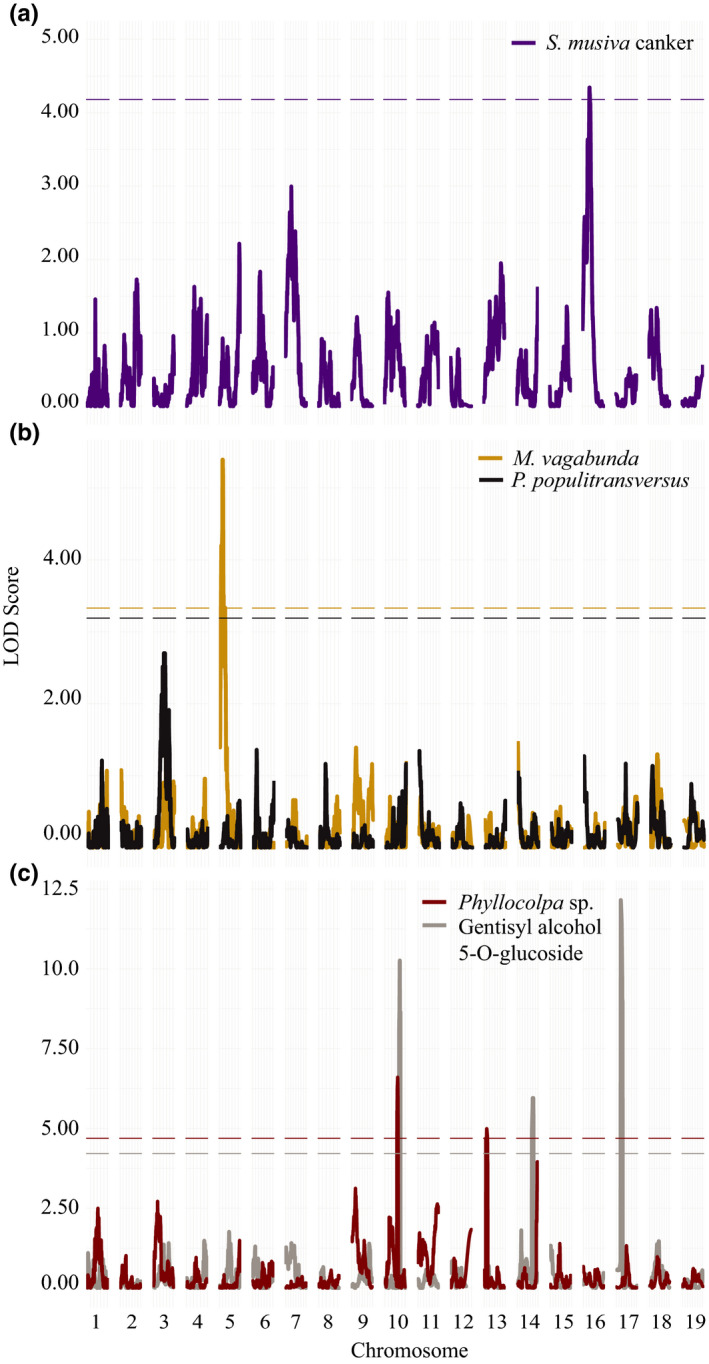
QTL interval plots showing peaks across the genome that associate with biotic surveys. Lines on the plots indicate *p*‐value thresholds as determined by running 1,000 permutations of mapping model for (a) binary fungal survey of *S. musiva* canker, (b) binary insect surveys of *M. vagabunda* and *P. populitransversus*, (c) and insect surveys of *Phyllocolpa* sp. leaf gall counts and overlapping peaks for gentisyl alcohol 5‐O‐glucoside compound QTL

Upon exclusion of individuals that showed symptoms of the competing fungus, the strength of the association on Chr04 increased for *Melampsora* sp. (marker position marker position = 3.50687, *p*‐value = .0001), whereas the association was lost for *S. musiva* leaf spot, and a new association was revealed on Chr06 (marker position = 142.6761866, *p*‐value = .017). The top Chr04 marker explained 57.9% of variation in *Melampsora* sp. infection severity, while the new Chr06 association explained 4.77% of the variation in *S. musiva* leaf spot disease severity. The positive allele contributing to an increase in fungal infection on Chr04 was derived from *P. deltoides,* whereas the positive allele contributing to severity of *S. musiva* leaf spot infection on Chr06 was derived from *P. trichocarpa*. Binary presence of the canker symptoms caused by *S. musiva* (Figure 5a) was found to be associated with a QTL located on Chr16 (marker position = 60.81429, *p*‐value = .022). The positive allele contributing to increase in presence of *S. musiva* canker was from *P. trichocarpa*.

Three QTL intervals containing a total of 40 markers were significantly associated with insect abundance (Table 2, Figure 5b,c). A QTL on Chr05 (marker position = 21.83874, *p*‐value = .001) was associated with binary presence of *M. vagabunda* branch galls (Figure 5b). The positive allele contributing to an increase in presence of *M. vagabunda* was from *P. trichocarpa*. Two QTLs, one located on Chr10 (marker position = 57.69914343, *p*‐value = .015) and one located on Chr13 (marker position = 84.43857329, *p*‐value =0.045), were associated with the number of leaf‐folding galls from *Phyllocolpa* sp. (Figure 5c). The marker with the highest LOD score explained 9.65% of the variance in oviposition gall count, while 8.82% of the variance was explained by the top marker located on Chr13. For both markers, the positive allele contributing to an increase in the number of female oviposition galls was from *P. deltoides*. There were no QTL intervals that passed the permutation threshold for the binary presence of *P. populitransversus* petiole galls (Figure 5c).

### 3.3 QTL mapping of leaf metabolites

Only one tested compound, gentisyl alcohol 5‐O‐glucoside levels, yielded significant QTL. Three QTL intervals containing a total of 56 significant markers were significantly associated with gentisyl alcohol 5‐O‐glucoside levels (Table 2, Figure 5c). This included markers on chromosome 17 (marker position = 21.54739208, *p*‐value < .0001) which explained 25.8% of variation, Chr10 (marker position = 109.8469079, *p*‐value < .0001) which explained 23.1% of variation, and chromosome 14 (marker position = 91.51828521, *p*‐value = .0100) which explained 7.30% of variation. The positive allele that contributed to an increase in gentisyl alcohol 5‐O‐glucoside levels for all markers was derived from *P. trichocarpa*.

### 3.4 Interaction between *Melampsora* sp. and *S. musiva* leaf infection

**FIGURE 6:**
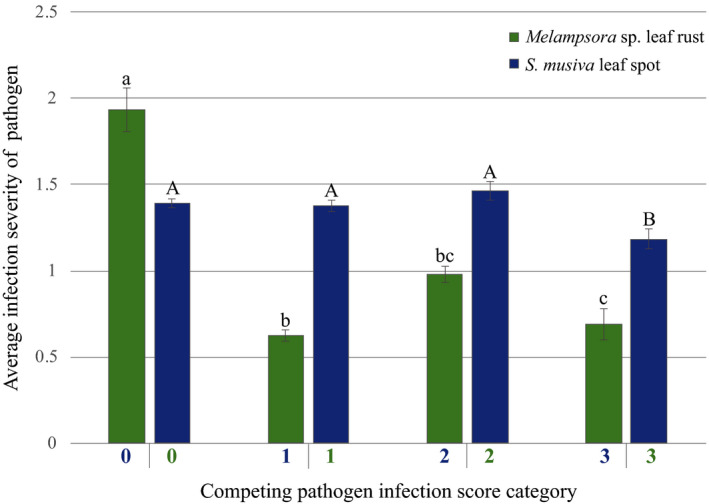
Mean and standard error of fungal infection for individuals with varying category levels of competing fungus infection. Letters indicate significantly different means as determined by Tukey’s honest significance test for each one‐way ANOVA test

*Melampsora* sp. leaf rust infection severity was dependent upon the disease severity of *S. musiva* leaf spot symptoms (*F* = 35.2, *p*‐value = .0001; Table 3). The severity of *Melampsora* sp. infection for individuals with no *S. musiva* leaf spot was significantly higher than for individuals with *S. musiva* leaf spot symptoms (Figure 6). Similarly, *S. musiva* leaf spot disease severity was dependent upon the levels of *Melampsora* sp. leaf rust (*F* = 31.5, *p*‐value = .0001). *S. musiva* leaf spot infection severity was significantly lower for individuals with a *Melampsora* sp. leaf spot score of 3 compared to individuals with less severe *Melampsora* sp. leaf spot symptoms (Figure 6).

**TABLE 3:** One‐way ANOVA, with genotype as a covariate, analyzing the effect of infection severity of the genus of one fungus on the infection severity of the competing leaf fungi in 2008

Factor	*df*	Sum of squares	Mean square	*F*‐ratio	*p*‐Value
Dependent variable—*Melampsora* sp. leaf rust infection					
Genotype	732	1,120	1.53	4.69	<.0001
*S. musiva* score	3	34.4	11.5	35.2	<.0001
Residuals	602	197	0.326		
Dependent Variable—*S. musiva* leaf spot infection					
Genotype	732	379	0.518	1.77	<.0001
*Melampsora* sp. score	3	27.6	9.19	31.5	<.0001
Residuals	602	176	0.292		

### 3.5 PFAM, GO term and tandem duplication enrichment

The total number of genes present in the QTL intervals of each parental species was 174 on Chr04, 81 on Chr16, 161 on Chr10, 445 on Chr13, and 156 on Chr05 (Table 4). There were 40 PFAM domains enriched relative to the rest of the *P. trichocarpa* genome across all QTL intervals (alpha = 0.05; *p*‐value threshold < .00014) and 25 PFAM domains enriched in the *P. deltoides* QTL intervals (alpha = 0.05, *p*‐value < .000059; Table S2). Additionally, there were 21 GO terms enriched in the *P. trichocarpa* intervals (alpha = 0.05, *p*‐value < .000226) and 46 GO terms enriched in the *P. deltoides* intervals (alpha = 0.05, *p*‐value < .000214; Table S3). Finally, seven of the genes in these intervals showed evidence of positive selection based on the ratio of nonsynonymous to synonymous nucleotide substitutions between the *P. deltoides* and *P. trichocarpa* orthologs (Table 5).

**TABLE 4:** Number of genes in QTL intervals in parental genomes for biotic associations

Fungus/insect	QTL interval	# Genes in *P. trichocarpa*	# Genes in *P. deltoides*
*Melampsora* sp.	Chr04	159	147
*S. musiva* canker	Chr16	71	68
*S. musiva leaf spot*	Chr06	129	132
*Phyllocolpa* sp.	Chr10	146	154
*Phyllocolpa* sp.	Chr13	252	425
*M. vagabunda*	Chr05	129	147

**TABLE 5:** Candidate genes under positive selection (Ka/Ks > 1) in genetic intervals associated with fungi and insects

QTL	Arabidopsis function	*P. trichocarpa* gene	*P. deltoides* gene	Ka/Ks
Chr04	Chitin elicitor receptor kinase 1	Potri.004G005800	Podel.04G004900.1.p	1.35
Chr04	Cysteine‐rich RLK (RECEPTOR‐like protein kinase)	Potri.004G012600	Podel.04G010900.1.p	2.23
Chr04	Stigma‐specific Stig1 family protein	Potri.004G006800	Podel.04G005700.1.p	1.31
Chr04	Stigma‐specific Stig1 family protein	Potri.004G007200.1	Podel.04G006100.1.p	1.00
Chr04	Stigma‐specific Stig1 family protein	Potri.004G007200	Podel.04G006100.1.p	1.00
Chr05	Pentatricopeptide repeat (PPR) superfamily protein	Potri.005G038500.1	Podel.05G042200.1.p	1.52
Chr10	Copper amine oxidase family protein	Potri.010G088700	Podel.10G084500.1.p	1.19

**FIGURE 7:**
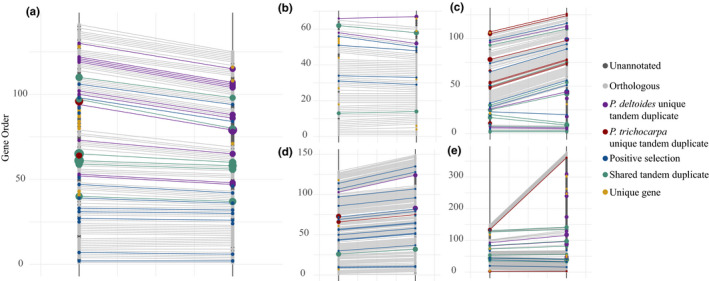
Comparison of gene content in *P. trichocarpa* grandparent 93‐968 (left line) and *P. deltoides* grandparent ILL‐101 (right line) for significant genetic intervals for (a) *Melampsora* sp. Chr04, (b) *S. musiva* Chr16, (c) *M. vagabunda* Chr05, (d) *Phyllocolpa* sp. Chr10, and (e) *Phyllocolpa* sp. Chr13 associations. QTL intervals were defined as 1Mb regions centered on the marker with the highest LOD score. Size of gene point is relative to the number of genes in the tandem duplication expansion

In total, there were 107 recent tandem duplicates in the *P. trichocarpa* intervals and 195 in the *P. deltoides* intervals for all biotic QTLs (Figure 7, Table 6). This includes 23 and 41 recent tandem duplicates for the *Melampsora sp.* intervals; 6 and 10 for the *S. musiva* intervals; 32 and 96 for the *Phyllocolpa* sp. intervals; and 47 and 46 for the *M. vagabunda* intervals, for *P. trichocarpa* and *P. deltoides*, respectively. The total number of recent tandem duplicates was significantly enriched relative to tandem counts for random intervals of the same size as the QTL intervals for the *P. deltoides* grandparent (*p*‐value = .0118) but not for the *P. trichocarpa* grandparent (*p*‐value = .1191).

**TABLE 6:** Tandem duplication profiles for genetic intervals. Number of copies next to species gene name indicates the size of tandem expansion for the gene

Arabidopsis function	*P. trichocarpa* gene	Copy #	*P. deltoides* gene	Copy #	QTL Chr
ERD (early‐ responsive to dehydration stress) family protein	Potri.004G005900	3	Podel.04G005000.1.p	3	Chr04
Pentatricopeptide repeat (PPR) superfamily protein	NA	0	Podel.04G010100.1.p	2	Chr04
Peroxidase superfamily protein	Potri.004G015300	1	Podel.04G013500.1.p		Chr04
Disease resistance protein (TIR‐NBS‐ LRR class)	Potri.005G031900	7	Podel.05G035500.1.p	12	Chr05
Shikimate O‐Hydroxycinnamoyl‐transferase	Potri.005G028000	3	Podel.05G029500.1.p	2	Chr05
Protein tyrosine kinase (Pkinase_Tyr) Leucine‐rich repeat (LRR_8)	Potri.005G030600	4	Podel.05G030600.1.p	2	Chr05
Lipoxygenase	Potri.005G032400	4	Podel.05G037200.1.p	3	Chr05
Cytochrome P450, family 721, subfamily A, polypeptide 1	Potri.005G034500	2	Podel.05G039300.1.p	1	Chr05
Cytochrome P450, family 76, subfamily G, polypeptide 1	Potri.005G029200	5	Podel.05G034500.1.p	0	Chr05
Cytochrome P450, family 93, subfamily D, polypeptide 1	Potri.005G037100	2	NA	0	Chr05
Cytochrome P450, family 76, subfamily G, polypeptide 1	NA	0	Podel.05G034500.1.p	4	Chr05
Disease resistance protein (TIR‐NBS class), putative	Potri.005G032000	1	Podel.05G035800.1.p	12	Chr05
Receptor‐like kinase in flowers	Potri.005G040200	1	Podel.05G043500.1.p	2	Chr05
Pentatricopeptide repeat (PPR) superfamily protein	Potri.010G083800	3	Podel.10G079300.1.p	3	Chr10
Copper amine oxidase family protein	Potri.010G088800	3	NA	0	Chr10
Ankyrin repeat family protein	Potri.013G133400	5	Podel.13G142400.1.p	10	Chr13
O‐methyltransferase family protein	Potri.013G143700	4	Podel.13G181100.1.p	1	Chr13
O‐methyltransferase family protein	NA	0	Podel.13G144000.1.p	4	Chr13
G‐type lectin receptor‐like protein kinase	Potri.016G102500	4	Podel.16G106900.1.p	4	Chr16

## 4 DISCUSSION

The goal of our research was to utilize QTL analysis as a tool to identify regions of the *Populus* genome that were important in mediating biotic interactions. Upon identification of these regions, we were able to directly compare the parental genomes of the hybrid cross to look for similarity in content and potential gene‐for‐gene interactions reflected in recent tandem duplication expansion. We found that the host plant genotype had a significant effect on fungi and insects in our study. Additionally, the progeny segregated for varying resistance to fungal and insect pathogens and pests that were inherited from the grandparents and parents of the 52–124 family cross.

Novel alleles (i.e., those from the grandparent that was not native to the region where the trials occurred) appear to be important for resistance to *Melampsora* sp. fungus, *M. vagabunda* galls, and *Phyllocolpa* sp. oviposition. This is not the first case of potential novel alleles being important in resistance to biotic stress. In a *P. trichocarpa* GWAS population, three genes have been found that confer novel resistance to *S. musiva* canker infection and an additional single gene that, when inherited, can suppress that resistance (Muchero et al., 2018). Although cases such as this are still under investigation to understand mechanisms of novel resistance, it can also originate from a variety of simple traits inherent to a non‐native species such as delayed emergence due to phenology (Mercader, Aardema, & Scriber, 2009) or changes in secondary metabolites that are important cues in insect recognition of the host important in oviposition (Nahrstedt, 1989). In contrast, the interval associated with *S. musiva* canker symptoms was the only case in which susceptibility to the fungus was dominant and inherited from the noncoevolved host (*P. trichocarpa*), as has been previously observed (Muchero et al., 2018; Newcombe & Ostry, 2001).

We detected a major QTL for *Melampsora sp.* resistance on Chr04. Although the specific strain that infected the trees is unknown, this genomic interval is known to contain the *MXC3* locus which confers resistance to infection of multiple species of the *Melampsora* leaf rusts (Newcombe, Stirling, & Bradshaw, 2001; Yin, DiFazio, Gunter, Jawdy, et al., 2004). Based on mean parental infection scores and the allelic effects at the QTL, progeny in family 52–124 inherited this resistance from the *P. trichocarpa* grandmother 93–968. Furthermore, the QTL interval contained two tandem repeats of stigma‐specific proteins (Stig1) in both the *P. trichocarpa* genome and the orthologous interval in the *P. deltoides* genome. Several of these genes showed evidence of positive selection based on Ka/Ks ratios (Table 5). Stigma‐specific proteins, specifically Stig1, have been found to be associated with female sterility in tobacco (*Nicotiana tabacum*) and petunia (*Petunia hybrida*; Goldman, Goldberg, & Mariani, 1994; Verhoeven et al., 2005). Stig1 is known to mediate secretion of exudate lipids in the intercellular spaces and high expression of the protein inhibits pollen grains from penetrating style tissue preventing fertilization (Verhoeven et al., 2005). Most lipid transfer proteins, such as Stig1, are important in plant cell wall loosening, and their expression can prevent penetration of plant tissues (Nieuwland et al., 2005). Diversification of the protein family containing Stig1 may play an important role in lowering fungal infection in *Populus* by providing a physical barrier to resist the *Melampsora* sp. hyphae. Another protein domain that was found to be enriched in the *P. trichocarpa* Chr04 interval was the malonyl‐CoA decarboxylase C‐terminal domain. Similarly, the gene ontology function for malonyl‐CoA decarboxylase activity was also enriched in *P. trichocarpa* in the same interval. Genes which are capable of transforming malonyl‐CoA could be beneficial in resistance to *Melampsora* sp. as it is an important precursor in the production of several pathogen defensive compounds, such as isoprenoids in the Mevalonate pathway (Chen, Kim, Weng, & Browse, 2011; Dixon, 2001).

Interestingly, an overlapping interval on Chr04 was found to be associated with the activity of the leaf spot symptoms of the *S. musiva* fungus. Upon further investigation, we found that trees that were not infected by the *S. musiva* leaf spot had more severe symptoms of the *Melampsora* sp. leaf rust. This suggests that a competitive interaction occurred between the two pathogens in the field during the year of survey and was reflected in an inflated association on Chr04 with the *S. musiva* leaf spot score. Furthermore, when individuals with presence of *Melampsora* sp. infection were removed from the *S. musiva* analysis, the association with Chr04 is no longer significant, and a new QTL appeared on Chr06 (Table S5). Competition among fungal pathogens is not uncommon in field conditions with most examples focused on different genotypes within the same fungal species (Abdullah et al., 2017). The outcome of within host tissue colonization of multiple strains or species often relies upon the genetic similarity of the pathogens (Abdullah et al., 2017; Koskella, Giraud, & Hood, 2006). In our case, we had two very different pathogens that were utilizing the leaf tissue in vastly dissimilar ways. *S. musiva* is a necrotrophic fungus, which requires dead tissue to reproduce, and *Melampsora* sp. is biotrophic, requiring living tissue to reproduce. The presence of *S. musiva* on the trees had a much larger effect on the occurrence of *Melampsora* sp. infection. This may indicate that the *S. musiva* fungus had a competitive advantage over *Melampsora* sp. in utilization of the *Populus* leaf tissue at our site. Further supporting this finding was the complete loss of *Melampsora* sp. symptoms at the plantation site over the course of ten years and the continual presence of the *S. musiva* leaf spot. A similar interaction has been reported in wheat between the biotroph *Blumeria graminis* f.sp. *tritici* (*Bgt*), the powdery mildew pathogen, and necrotroph *Zymoseptoria tritici*, the cause of *Septoria tritici* blotch (Orton & Brown, 2016). Similarly, the outcome of the interaction of the two pathogens in wheat was competitive. However, the necrotroph was found to actually be capable of reducing the reproductive capability of the biotroph which indicates pathogen–pathogen interactions can be more direct rather than relying solely on the host plant genetics (Orton & Brown, 2016).

The *S. musiva* leaf spot and stem canker symptoms were found to be associated with QTL intervals on Chr06 and Chr16, respectively. In this study, susceptibility to the necrotrophic fungi appeared to be originating from the presence of *P. trichocarpa* alleles in the progeny for both symptoms. Previous work on a *P. trichocarpa x deltoides* intercross supports this with susceptibility to *S. musiva* necrotrophic fungi originating primarily from dominant alleles derived from *P. trichocarpa* (Newcombe, 1998; Newcombe & Ostry, 2001). The Chr06 leaf spot association contained numerous genes with methyltransferase activity which was higher in the *P. deltoides* interval. Methyltransferase enzymes are important in plant secondary metabolism and have been found to be key in the production of a variety of antimicrobial compounds (Noel, Dixon, Pichersky, Zubieta, & Ferrer, 2003). Interestingly, the loci conferring susceptibility in family 52–124 in the Chr16 canker interval did not overlap with the four loci that were uncovered in a previous genome‐wide association study of *S. musiva* susceptibility in *P. trichocarpa* (Muchero et al., 2018), suggesting that different mechanisms may be involved in hybrid interactions with this pathogen. However, the QTL did contain a tandem repeat of a G‐type lectin receptor‐like protein kinase that was expanded in *P. deltoides*. This protein could play a similar role to a receptor‐like kinase from the same family (Yang et al., 2016) that was associated with susceptibility to *S. musiva* in the *P. trichocarpa* study (Muchero et al., 2018).

*M. vagabunda* has been recorded completing its life cycle on several species of *Populus* including *P. deltoides* and *P. tremuloides* (Floate, 2010). Although the aphid's life history has been documented, little is known about the influence of host plant genetics on gall formation or resistance to feeding (Floate, 2010; Ignoffo & Granovsky, 1961a). We detected a QTL on Chr05 in which alleles inherited from *P. deltoides* were positively associated with gall occurrence. Genes conferring lipoxygenase and oxidoreductase activity were enriched in both the *P. deltoides* and the *P. trichocarpa* QTL intervals. Lipoxygenase genes are known to be associated with the *Populus* response to both abiotic and biotic stressors (Cheng et al., 2006; Ralph et al., 2006). They are often upregulated in the presence of mechanical damage, fungal pathogen invasion, and exposure to simulated insect feeding (Chen, Liu, Tschaplinski, & Zhao, 2009; Cheng et al., 2006). The lipoxygenases are important in the formation of jasmonic acid, the signaling molecule that upregulates plant defenses against herbivore feeding (Chen et al., 2009).

The *M. vagabunda* QTL interval on Chr05 also contained a tandem array of resistance genes (R‐genes) that encoded disease resistance proteins (TIR‐NBS‐LRR class) that were greatly expanded in *P. trichocarpa* compared to *P. deltoides*, as well as repeats of the leucine‐rich repeat protein kinase family proteins in both species. These protein families are well known for their roles in the recognition and upregulation of host plant defenses against bacterial and fungal infection (Bergelson et al., 2001; Martin, Bogdanove, & Sessa, 2003). Neofunctionalization of R‐genes through tandem duplication due to gene‐for‐gene coevolution has also been demonstrated in many plant–fungal pathosystems (Leister, 2004). Although R‐genes have been more frequently related to plant–fungal interactions, there is increasing evidence that they are also important in mediating plant–insect interactions, especially in insects that utilize piercing‐sucking feeding (Harris et al., 2003; Kaloshian, 2004).

In addition to the R‐genes, there were a series of genes encoding cytochrome P450 family proteins in both species and a unique tandem set that was only present in the *P. trichocarpa* genome. P450 enzymes are important in the production of many classes of secondary metabolites such as furanocoumarins and terpenoids which are highly toxic to insects (Keeling & Bohlmann, 2006; Schuler, 2011). Alternatively, cytochrome P450’s may also be implicated in the susceptibility of the host plant to galling aphids. They are important in the synthesis of fatty acids and production of suberin in plant tissues (Höfer et al., 2008; Pinot & Beisson, 2011). Typically, suberin is important in separation of different tissues as well as in the establishment of apoplastic barriers that restrict nutrient/water loss as well as pathogen invasion (Höfer et al., 2008; Qin & LeBoldus, 2014). Several insects are known to produce suberized spherical galls on leaves including hymenopteran pests of Rosacea species and dipteran pests of Fabaceae (Krishnan & Franceschi, 1988; Oliveira et al., 2016). If aphids induce the suberization mechanism of the plant genome, it may lead to the increased toughening of aphid galls, much like the woody structures *M. vagabunda* leaves behind on branches once they have finished feeding.

We detected two QTL for *Phyllocolpa* sp. with *P. trichocarpa* alleles being positively associated with leaf fold occurrence in both cases. The *P. trichocarpa* genomic interval corresponding to the Chr10 QTL was enriched for several domains and GO terms that could be involved in gall development. For example, the interval was enriched for sugar transporters as well as GO terms for sucrose transporter activity. Often in the case of galling insects, plant tissue is modified in such a way as to act as a sugar sink, thereby enhancing its nutritional value to larvae (Larson & Whitham, 1991; Nyman, Widmer, & Roininen, 2000; Wool, 2004). The presence of these combinations of sugar transporter genes may be mediating a similar interaction between the *Phyllocolpa* sp. female sawflies and their chosen *Populus* hosts.

*Phyllocolpa* sp. galls are formed early in the season when a female sawfly selects a leaf and injects the longitudinal fold with small amounts of fluid on the underside of young leaves (Fritz & Price, 1988; Kopelke, 2007). The adult sawfly will proceed to oviposit near the base of the leaf and after 1–2 days the leaf fold forms, and the newly hatched larvae feeds on the inside of the gall (Smith & Fritz, 1996). The *Phyllocolpa* sp. galls were a unique biotic phenotype to this study as they were an estimate of female sawfly ovipositional choice rather than feeding success. Host selection for oviposition is initially driven by visual cues and reinforced by females assessing the nutrition and chemical cues of foliage (Boeckler, Gershenzon, & Unsicker, 2011; Panda & Khush, 1995). Previous research in *Salix* (closely related to *Populus*) has shown that common phenolic glycosides in leaf tissue are important in host choice in both the free‐feeding *Nematus oligospilus* and galling *Euura amerinae* specialist sawflies (Fernández et al., 2019; Kolehmainen, Roininen, Julkunen‐Tiitto, & Tahvanainen, 1994). We therefore pursued QTL mapping of metabolites to determine whether phenolic compounds might be important determinants of the potential ovipositional relationship.

We detected three significant QTL for gentisyl alcohol 5‐O‐glucoside levels. One of these overlapped with the *Phyllocolpa* sp. leaf fold QTL on Chr10. The QTL on Chr14 also overlapped with a suggestive QTL peak for *Phyllocolpa sp*. (LOD = 3.96). This is the first case of an association of gentisyl alcohol 5‐*O*‐glucoside with a specialist arthropod in *Populus*, and is, in fact, the first report of this metabolite in *Populus*. Salirepin (gentisyl alcohol 2‐*O*‐glucoside), a closely related metabolite, is a well‐known constituent in *Populus* sp (Busov et al., 2006; Tschaplinski et al., 2019; Veach et al., 2018), and leaves of *P. deltoides* and *P. trichocarpa x deltoides* have a lower abundance, later eluting metabolite with a nearly identical fragmentation pattern to salirepin that we tentatively identify as gentisyl alcohol 2‐*O*‐glucoside. The QTL contains three putative candidate genes, including an aldehyde dehydrogenase 5F1 (Podel.10G175800) that may be involved in the reduction of gentisyl aldehyde to the alcohol, and two UDP‐glycosyltransferases (Podel.10G184800, Podel.10G185000) that may be involved in the gentisyl alcohol conjugation to glucose. Specific substrates have yet to be determined for these genes, but a previous report suggests aldehyde dehydrogenase 5F1 genes are likely involved in the basic metabolism of *Populus* (Tian et al., 2015).

*Phyllocolpa* sp. sawflies are considered a keystone species as the abandoned or unused leaf folds are often used as a habitat for many other species such as aphids and spiders (Bailey & Whitham, 2007). The presence of folds in aspen forests is associated with a twofold increase in arthropod species richness and around a fourfold increase in arthropod abundance relative to forests where the insect is absent (Bailey & Whitham, 2003). This in turn makes the host plant and sawfly relationship important in examining how shifts in the genes of a population ultimately structure whole communities, effectively linking ecology and evolutionary biology. Further investigation of this potential relationship could be a key to connecting *Populus* genetics to the assemblage of the surrounding communities of organisms.

A striking finding in this study was an elevated number of recent tandem duplications in the *P. deltoides* genome but not the *P. trichocarpa* genome for the biotic QTL intervals. Out of the six chromosomes that yielded significant QTL results, four were associated with phenotypes that were fungi and insects native to the distribution of *P. deltoides*, but not *P. trichocarpa*. Given that *P. deltoides* has been coevolving with the majority of the surveyed fungi and insects, it was not unexpected that there were more recent tandem duplicates in biotic intervals in its genome as there is more selective pressure on the native species to overcome biotic stress (Constabel & Lindroth, 2010; Newcombe, Martin, & Kohler, 2010). However, given the high amount of novel resistance occurring in the progeny, recent tandem duplication may also be important in naïve host resistance.

In our study, we demonstrated how host plant genetics directly affect associated fungi and insects in the field, as well as how *Populus* progeny indirectly structured interactions between pathogens (Whitham et al., 2006). The competition between *Melampsora* sp. and *S. musiva* highlights the complexities of how hybrid genetics are capable of strongly mediating multiple species interactions, which can result in inflated genetic associations. Finally, we have shown that many recent tandem duplications, found across biotic stress QTL intervals, have functional annotations that are involved in host plant physical/chemical resistance and tolerance as well as a few that may be implicated in host plant susceptibility. The enrichment of recent tandem duplications is a signature of gene‐for‐gene interactions and a mechanism that is essential to protect long‐lived plants such as trees, enabling them to reach maturity despite many coevolving biotic stressors.

## CONFLICT OF INTEREST

None declared.

## AUTHOR CONTRIBUTION

**Sandra J. Simon:** Conceptualization (equal); Data curation (supporting); Formal analysis (equal); Investigation (lead); Methodology (supporting); Writing‐original draft (lead); Writing‐review & editing (supporting). **Timothy J. Tschaplinski:** Formal analysis (supporting); Investigation (supporting); Methodology (supporting); Writing‐original draft (supporting); Writing‐review & editing (supporting). **Jared Leboldus:** Data curation (supporting); Investigation (supporting); Methodology (supporting); Writing‐original draft (supporting); Writing‐review & editing (supporting). **Ken Keefover‐Ring:** Data curation (supporting); Formal analysis (supporting); Investigation (supporting); Writing‐original draft (supporting); Writing‐review & editing (supporting). **Muhammad Azeem:** Formal analysis (supporting); Investigation (supporting). **Jin‐Gui Chen:** Data curation (supporting); Funding acquisition (supporting); Investigation (supporting); Writing‐review & editing (supporting). **David Macaya‐Sanz:** Data curation (supporting); Formal analysis (supporting); Investigation (supporting). **William L. MacDonald:** Data curation (supporting); Investigation (supporting); Methodology (supporting). **Wellington Muchero:** Conceptualization (supporting); Funding acquisition (supporting); Investigation (supporting); Methodology (supporting). **Stephen P. DiFazio:** Conceptualization (equal); Formal analysis (supporting); Funding acquisition (supporting); Investigation (equal); Methodology (equal); Project administration (equal); Writing‐original draft (supporting); Writing‐review & editing (lead).

## Supporting information

Table S1‐S5

## Data Availability

DNA sequences are deposited at NCBI Sequence Read Archive under the following accession numbers: SRP112750, SRP109803, and SRP190316. The annotated *P. deltoides* clone WV94 genome assembly is available through Phytozome (https://phytozome.jgi.doe.gov/pz/portal.html).
